# Management of acquired resistance to EGFR TKI–targeted therapy in advanced non-small cell lung cancer

**DOI:** 10.1186/s12943-018-0777-1

**Published:** 2018-02-19

**Authors:** Shang-Gin Wu, Jin-Yuan Shih

**Affiliations:** Department of Internal Medicine, National Taiwan University Hospital, No. 7, Chung-Shan South Road, Taipei, 100 Taiwan

## Abstract

Recent advances in diagnosis and treatment are enabling a more targeted approach to treating lung cancers. Therapy targeting the specific oncogenic driver mutation could inhibit tumor progression and provide a favorable prognosis in clinical practice. Activating mutations of epidermal growth factor receptor (*EGFR*) in non-small cell lung cancer (NSCLC) are a favorable predictive factor for EGFR tyrosine kinase inhibitors (TKIs) treatment. For lung cancer patients with *EGFR*-exon 19 deletions or an exon 21 Leu858Arg mutation, the standard first-line treatment is first-generation (gefitinib, erlotinib), or second-generation (afatinib) TKIs. EGFR TKIs improve response rates, time to progression, and overall survival. Unfortunately, patients with *EGFR* mutant lung cancer develop disease progression after a median of 10 to 14 months on EGFR TKI. Different mechanisms of acquired resistance to first-generation and second-generation EGFR TKIs have been reported. Optimal treatment for the various mechanisms of acquired resistance is not yet clearly defined, except for the T790M mutation. Repeated tissue biopsy is important to explore resistance mechanisms, but it has limitations and risks. Liquid biopsy is a valid alternative to tissue re-biopsy. Osimertinib has been approved for patients with T790M-positive NSCLC with acquired resistance to EGFR TKI. For other TKI-resistant mechanisms, combination therapy may be considered. In addition, the use of immunotherapy in lung cancer treatment has evolved rapidly. Understanding and clarifying the biology of the resistance mechanisms of *EGFR*-mutant NSCLC could guide future drug development, leading to more precise therapy and advances in treatment.

## Background

In the United States, an additional 224,390 new lung cancer cases were diagnosed in 2016, and accounted for about 27% of all cancer deaths [1]. Although standard platinum-based chemotherapy is the cornerstone of systemic therapy, it has a modest effect on overall survival (OS) [2]. Lung cancer is still the leading cause of cancer death worldwide [3].

In the most recent decade, treatment of non-small cell lung cancer (NSCLC) has evolved to a great extent. The discovery of driver mutations in lung cancer allows the creation of personalized targeted treatment. It is important that lung cancer patients are tested for oncogenic drivers of cancer and receive matched targeted therapy [4]. Epidermal growth factor receptor tyrosine kinase inhibitors (EGFR TKIs) provide a favorable treatment outcome in epidermal growth factor receptor (*EGFR*) mutation-positive patients. *EGFR* mutation-positive patients with lung adenocarcinoma had a response rate as high as 80%, and around 10–14 months of progression-free survival (PFS) [5, 6]. The American Society of Clinical Oncology (ASCO), European Society for Medical Oncology (ESMO) and National Comprehensive Cancer Network (NCCN) guidelines recommend EGFR TKIs as first-line treatment for *EGFR*-mutant patients. The most common activating mutations are in-frame deletions in exon 19 and single-point mutation of exon 21 (Leu858Arg), which together account for more than 80% of known activating *EGFR* mutations [7, 8].

Although EGFR TKIs have a favorable and durable treatment response, most patients will eventually develop progressive disease (PD) within about one year of treatment. Furthermore, acquired resistance develops and limits the long-term efficacy of these EGFR TKIs. A variety of mechanisms of acquired resistance to EGFR TKIs have been reported. The most common mechanism is the development of acquired *EGFR* T790M mutation [9]. T790M was found in about 50% of *EGFR*–mutant cases that acquired resistance to EGFR TKIs [9]. Patients using either first- or second-generation EGFR TKIs had a similar prevalence of acquired T790M [10].

Preclinical data showed that the second-generation EGFR TKI, afatinib, could overcome the resistance caused by the T790M mutation [11], but clinical trials have not revealed the effect due to toxicity limitations. The narrow therapeutic window of afatinib caused severe adverse effects (AEs), probably owing to inhibition of wild-type *EGFR* [12, 13]. In the phase III LUX-Head & Neck 1 (LHN1) trial, second-line afatinib significantly improved PFS versus methotrexate in patients with recurrent/metastatic head and neck squamous cell carcinoma [14]. This suggests afatinib is a drug active against wild-type *EGFR*. The third-generation EGFR TKI, osimertinib, has been approved for patients with T790M-positive NSCLC with acquired resistance to EGFR TKIs. Use of third-generation EGFR TKIs was related to different acquired resistance mechanisms [15–18]. Therefore, in this manuscript, we focused on these recently developed treatment strategies for *EGFR*-mutant NSCLC with acquired resistance to first- or second-generation EGFR TKIs.

### Clinical presentation of acquired resistance to first-line EGFR TKIs

Although *EGFR*-mutant patients receiving EGFR TKIs have longer median PFS than those receiving platinum-based chemotherapy as first-line treatment [5, 6, 19, 20], acquired resistance to EGFR TKIs eventually emerges. In 2010, Jackman et al. proposed clinical criteria for acquired resistance to EGFR TKI based on the Response Evaluation Criteria in Solid Tumors (RECIST) [21, 22]. Acquired resistance is defined as when *EGFR*-mutant NSCLC patients achieved a response or stable disease with greater than six months of targeted therapy and subsequently developed disease progression while still on the targeted agent [22]. However, the patterns of disease progression varied in clinical practice.

Oncologists traditionally change treatment regimens when there is objective evidence of radiological or clinical progression. However, in routine practice, different characteristics of disease progression might develop when using EGFR TKIs, and will confuse clinicians. Gandara et al. divided disease progression with EGFR TKIs use into three subtypes, including: oligoprogression (new sites or regrowth in a limited number of areas, maximum of four progression sites), systemic progression (multisite progression), and central nervous system (CNS) sanctuary progression (excluding leptomeningeal carcinomatosis due to the lack of effective treatment options for long-term control) [23]. For patients with CNS sanctuary progression and/or oligoprogressive disease when using a previously beneficial EGFR TKI, it may be reasonable to consider local treatment and continuation of the targeted agent. This approach yielded more than six months of additional disease control [24, 25].

Yang et al. proposed another criteria for EGFR TKI failure modes in NSCLC [26]. Based on the duration of disease control, the evolution of the tumor burden, and clinical symptoms, regardless of genotype profile, the diversity of EGFR TKI failure could be categorized into three modes, including dramatic progression, gradual progression, and local progression. The median PFS was 9.3, 12.9, and 9.2 months (*p* = 0.007) for these three modes, respectively, and median OS was 17.7, 39.4, and 23.1 months (*p* < 0.001), respectively. In patients with disease in the gradual progression mode, continuing EGFR TKI therapy was superior to switching to chemotherapy in terms of OS (39.4 vs. 17.8 months; *p* = 0.02) [26]. Determination of the clinical mode could favor strategies for subsequent treatment and prediction of survival.

### Mechanisms of acquired resistance to EGFR TKIs

**Fig. 1:**
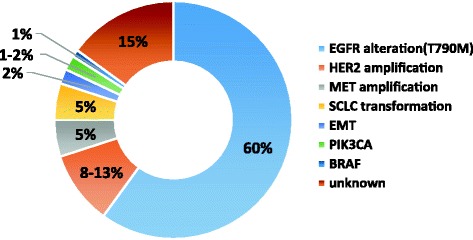
The distribution of different acquired resistance mechanisms

Acquired resistance mechanisms vary. Several study groups comprehensively explored the mechanisms through re-biopsy tissue specimens. The most common acquired resistance mechanisms were of three types: target gene modification, alternative pathway activation and histological or phenotypic transformation (Fig. 1).

#### Target gene modification

The T790M mutation, which substitutes methionine for threonine at amino acid position 790 at exon 20 of *EGFR*, was the most commonly acquired resistance mechanism. It accounted for about 50–60% of cases with acquired resistance to gefitinib or erlotinib [9, 10]. The 790 residue is in a key location at the entrance to a hydrophobic pocket of the ATP-binding cleft, so it is also referred to as a “gatekeeper” mutation. Because of the bulky methionine sidechain, T790M causes conformational change that leads to the development of steric hindrance and affects the ability of EGFR TKI to bind to the ATP-kinase pocket [9]. In addition, the T790M mutation of *EGFR* could restore the affinity of the mutant receptor for ATP, thus reducing the potency of competitive inhibitors [27].

Other second-point mutations, such as D761Y [28], T854A [29], or L747S [30], confer acquired EGFR TKI resistance, although the definite mechanism is still unclear.

#### Alternative pathway activation

Alternative or bypass pathway activation also causes primary resistance. Through bypass tract activation, cancer cells can survive and proliferate, even when inhibits by the initial driver pathway. The most common bypass pathway is *MET* amplification, which accounts for 5–10% of cases with acquired resistance to EGFR TKIs [31, 32]. *MET* gene amplification could activate PI3K-AKT pathway signaling independent of *EGFR* through driving ERBB3 dimerization and signaling [31]. However, the threshold of *MET* amplification that would induce TKI resistance has not been clarified. Overexpression of hepatocyte growth factor, the ligand of MET oncoprotein, also promotes EGFR TKI resistance [33].

Activation of other alternative pathways, including *HER2* amplification [34], *PIK3CA* mutation [35], *BRAF* mutation, and increased expression of the receptor tyrosine kinase AXL, have been reported to promote acquired resistance to EGFR TKIs [36].

#### Histological and phenotypic transformation

About 5% of patients suffered from transformation from *EGFR*-mutant adenocarcinoma to small-cell lung cancer (SCLC) after acquired resistance to EGFR TKIs [35]. A possible theory is that the initial sample bias resulted in missing the preexisting SCLC component in the original tumor. However, the patient had a good treatment response and prolonged PFS [37], and the original activating *EGFR* mutations of adenocarcinoma persisted in the re-biopsy SCLC specimens [38, 39]. Recent studies disclosed that the SCLC transformation process is predisposed in adenocarcinoma by inactivation of Rb and p53 [40, 41]. In addition, evaluation of the RB1 and TP53 status of adenocarcinoma is predictive biomarker for SCLC transformation after TKI treatment [40, 41]. SCLC transformation arises from common progenitor cells of adenocarcinoma in response to EGFR TKI therapy [37].

Inappropriate induction of epithelial–mesenchymal transition (EMT) in tumor cells caused tumor invasion, metastasis, drug resistance, and stem cell properties [42, 43]. Many studies have shown that EMT is a mechanism of acquired resistance to EGFR TKIs. Different EMT transcription factors, including Slug, ZEB1, Snail, and AXL, changed with the development of acquired resistance to EGFR TKIs [42, 44]. EMT was reported in two (5%) re-biopsy tumors of 37 patients [35]. In terms of morphology, the cancer cells lost their epithelial features (e.g., E-cadherin expression) and transformed into spindle-like mesenchymal cells with a gain of vimentin [45].

### Exploring the resistance mechanism of EGFR TKIs

Different mechanisms can be detected in disease progression to EGFR TKIs [46]. It is important to identify the definite tumor resistance mechanism. Repeated tumor biopsy is a key factor for the subsequent treatment plan. Genotyping, whether for the existence of *EGFR* T790M mutations or other oncogenic alterations, is a crucial step in guiding future treatment, according to the current NSCLC guidelines [47, 48].

However, tumor heterogeneity appears in the primary tumor and in metastatic lesions. Intratumor and inter-metastases may have diverse clones with different oncogenic driver mutations or resistance mechanisms [49]. The resistant mutations may occur at a small clone of tumor cells and clonal evolution may develop during the treatment process, so molecular-based detection methods play an important role. Mutation-enriched or ultra-sensitive (defined as an analytic sensitivity below 1%) molecular-based detection methods should be considered [46, 50]. The guideline of the College of American Pathologists, International Association for the Study of Lung Cancer, and Association for Molecular Pathology recommends that the assay for the *EGFR* T790M resistant mutation is able to detect the mutation in as few as 5% of cells or less (assuming heterozygosity, a 2.5% mutant allele fraction) in clinical practice [50]. For traditional PCR-based methods, Sanger sequencing provided a sensitivity of only about 20%. Other highly sensitive PCR-based assays utilizing locked nucleic acids (LNAs) or peptide nucleic acids (PNAs) could reach 0.1–2% of analytical sensitivity [51]. Kinase fusions recently were reported as mechanisms of acquired resistance to EGFR TKIs [52]. Next-generation sequencing (NGS) is becoming the preferred method because it can provide high sensitivity to detect known and unknown mutations and genetic alterations.

Sometimes, it is difficult to obtain the re-biopsy tumor specimens because of the potential risks of invasive diagnostic procedures. Prospective studies showed that the success rate of repeated biopsy was 75–95%, and serious complications were detected in about 1% of cases [32, 53, 54]. Although repeated biopsy seems safe in clinical practice, it is still limited in use because of patient fear and physician preference. Therefore, obtaining serial biopsies from the same patient is rarely feasible during the NSCLC treatment course. In addition, the existence of intra-tumor heterogeneity influences tumor evolution, metastasis and resistance mechanisms in different ways, including somatic mutations, epigenetic change and post-transcriptional modification [55–57]. Therefore, there may be selection bias because a single snapshot biopsy specimen is not enough to accurately represent all the resistance mechanisms of different sites.

Liquid biopsy, on the other hand, could provide a source of information on the resistance mutations of the entire tumor landscape, compared with the single site sampled using conventional tumor tissue biopsy [58]. Cell-free circulating DNA (ctDNA) is adopted for noninvasive exploration of resistance mechanisms and tumor genetic alterations. ctDNA theoretically could provide a surrogate of the whole tumor genome of both primary and metastatic lesions. Different methodologies, with high sensitivity and detection of genetic number and type alteration, are being used for ctDNA testing (Table 1) [59]. The *EGFR* T790M mutation could be detected in plasma samples by highly sensitive genotyping methods, including next-generation sequencing, droplet digital polymerase chain reaction (ddPCR), and bead, emulsion, amplification and magnetics (BEAMing) assays [60–63]. The FDA has approved the Roche real-time PCR assay, cobas® *EGFR* Mutation Test v2, for detection of *EGFR* mutations in ctDNA in blood samples. Using ctDNA to detect mutations can produce a high positive predictive value. But, not all tumors shed ctDNA to the same degree, because of differences in tumor size, stage, location, vascularity, sites of metastatic disease and treatment history [64, 65]. Several studies found that up to 35% of patients with *EGFR* T790M might have false-negative plasma levels, compared with tissue biopsy [66, 67]. Therefore, if liquid biopsy shows a negative *EGFR* T790M mutation, tissue biopsy for confirmation is necessary [66].

**Table 1:** Sensitivity of detection of circulating tumor DNA (ctDNA)

Test	Detection	Analytic limitation	*EGFR* T790M mutation		Test Characteristics	Reference
			Sensitivity	Specificity		
MS	Known SNVs	1–10%	38.9% for del19/L858R	84.6% for del19/L858R	Quantitative	[122]
dHLPC	Known SNVs	1–5%	81.8% for sensitizing mutation	89.5% for sensitizing mutation	Provided information only of presence/absence of known mutations	[123, 124]
Cobas	Known SNVs	1–3%	61.4% (76.7% for del19/L858R)	78.6% (98.2% del19/L858R)	Semi-quantitative The only FDA approved ctDNA assay for detection of *EGFR* mutations	[70, 71]
Scorpion-ARMS	Known SNVs	1–3%	61.8%–85.7% for del19/L858R	94.3–100% for del19/L858R	Semi-quantitative	[72, 125]
HRMA	Known SNVs, indels,	0.1–10%	91.67% for sensitizing mutation	100% for sensitizing mutation	Rapid *EGFR* mutation screening	[126]
ddPCR	Known SNVs	> 0.1%	77% (74–82% for del19/L858R)	63% (100% for del19/L858R)	Quantitative Rapid turnaround time	[73]
BEAMing	Known SNVs, CNVs, SV	> 0.1–0.01%	70%	69%	Quantitative Detects complex alteration	[66]
NGS	Known/new SNVs, indels, CNVs, SV	0.01%–5%	93% (87–100% for del19/L858R)	94% (96–100% for del19/L858R)	Quantitative Profiles large gene panels Detects more complex alteration	[127–129]
PNA-PCR	Known SNVs, indel,	0.01%	78% for del19/L858R	100% for del19/L858R	Semi-quantitative	[130, 131]

*SNV* single nucleotide variant, *ctDNA* circulating tumor DNA, *ARMS* amplification refractory mutation system, *BEAMing* beads, emulsion, amplification and magnetics, *ddPCR* digital droplet polymerase chain reaction, *del* deletion, *indel* insertion/deletion, *FDA* US Food and Drug Administration, *NGS* next-generation sequencing, *CNVs* copy number variants, *SV* structure variants, *HRMA* high-resolution melting analysis, *dHLPC* denaturing high performance liquid chromatography, *MS* mass spectrophotometry (MS), *PNA-PCR* peptide nucleic acid-polymerase chain reaction

Serial analysis of ctDNA can track the molecular dynamic evolution of the tumor and monitor treatment response. However, the technological approach is not standardized because of the broad range of ctDNA isolation techniques, DNA analysis and quantification [65, 68].

### The management of progression during EGFR TKIs use

According to the NCCN guideline [48], subsequent therapy after progression with first-line EGFR TKIs includes different treatment recommendations, which have been plotted as an algorithm. For patients with sensitizing *EGFR* mutations who progress during or after first-line targeted therapy, recommended therapy depends on the acquired resistance mechanism and whether the progression is asymptomatic or symptomatic.

**Fig. 2:**
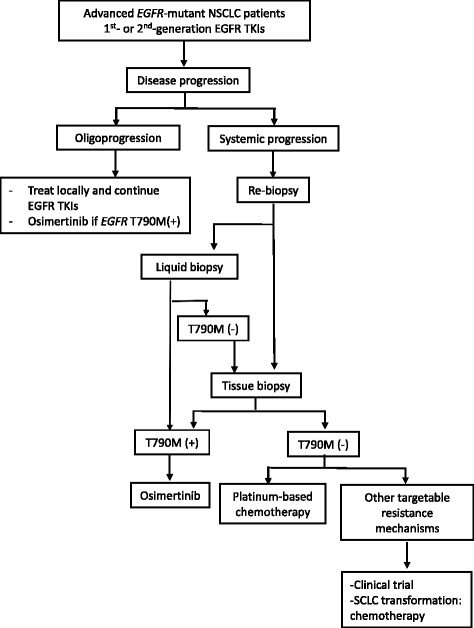
Treatment algorism for advanced *EGFR*-mutant NSCLC patients

We modified the latest NCCN and ESMO Guidelines [48, 69], and included the feasibility of liquid biopsy based on the emerging evidence from studies and trials [70–73]. An algorithm was proposed (Fig. 2) to provide clinical physicians with an appropriate practice plan for patients who experience disease progression on EGFR TKIs.

#### TKI beyond progression

In clinical practice, clinicians may prescribe EGFR TKI therapy beyond progression, especially when patients suffer from asymptomatic progression. Nishie et al. retrospectively analyzed Japanese patients with *EGFR* mutations. Continuous use of EGFR TKIs beyond progression in patients with activating *EGFR* mutations may prolong OS compared with switching to cytotoxic chemotherapy [74]. In addition, the phase II ASPIRATION study demonstrated that continued erlotinib therapy following progression is feasible in selected patients [75]. The NCCN Panel recommended continuing EGFR TKIs, whether erlotinib, gefitinib, or afatinib, and considering local therapy in patients with asymptomatic progression [48].

A flare-up phenomenon (rapid disease progression) occasionally is noted after discontinuation of EGFR TKIs. Intratumor heterogeneity is the possible mechanism of the phenomenon. Compared to the resistant clone with indolent behavior, rapid regrowth of TKI-sensitive clones causes rapid clinical deterioration when EGFR TKIs are discontinued [76]. One retrospective study also showed that 14 of 61 (23%) patients suffered from disease flare after stopping EGFR TKIs [77]. Therefore, some patients were prescribed EGFR TKIs after acquired resistance to EGFR TKIs.

The phase III IMPRESS trial aimed to evaluate the efficacy and safety of continuing gefitinib combined with chemotherapy versus chemotherapy alone in patients with *EGFR*-mutation-positive advanced NSCLC with acquired resistance to first-line gefitinib. A total of 265 patients were enrolled. However, continuation of gefitinib after disease progression on first-line gefitinib did not prolong PFS in patients treated with platinum-based doublet chemotherapy as subsequent treatment. A long-term follow-up found that median OS was 13.4 months in the combination arm and 19.5 months in the control arm (HR 1.44; *p* = 0.016) [78]. Besides, the gefitinib group had more side effects and grade 3 or worse AEs. According to the results of the IMPRESS trial, continuation of chemotherapy with first-generation EGFR TKIs after acquired resistance to EGFR TKIs is not considered as standard treatment.

#### Switch therapy

Repeated biopsy could provide information about the mechanism of acquired resistance. If there is no targetable oncogenic driver mutations/bypass pathways and corresponding target medications, chemotherapy is still the standard subsequent treatment after acquired resistance to EGFR TKIs. The NCCN guideline offers a treatment algorithm for patients whose disease has progressed on first-line EGFR TKIs. Platinum doublet with or without bevacizumab chemotherapy should be considered and recommended as second-line treatment for patients when they suffer from systemic progression due to acquired resistance to EGFR TKIs.

Two retrospective studies found that for *EGFR*-mutant patients who received platinum-based chemotherapy after disease progression with first-line EGFR TKI treatment, the response rates were 14–18%. Their median PFS with second-line chemotherapy was about four months [79, 80]. Because *EGFR* mutations are detected mostly in patients with an adenocarcinoma or non-squamous histology, the optimum regimen might be pemetrexed and platinum combination treatment [81], followed by maintenance pemetrexed for patients who did not suffer from disease progression [48, 82].

The most common mechanism of acquired resistance to EGFR TKIs is acquired T790M mutation. Second-generation EGFR TKIs, including afatinib, dacomitinib and neratinib, had efficacy in inhibiting proliferation of T790M mutation-positive cells in vitro*.* However, clinical trials showed disappointing results due to high toxicities resulting from the narrow therapeutic window. In contrast to second-generation EGFR TKIs, third-generation EGFR TKIs had a good treatment effect on tumors harboring *EGFR* T790M mutations [48, 83–85].

#### Next-generation (third-generation) epidermal growth factor receptor tyrosine kinases inhibitors (EGFR TKIs)

The third-generation EGFR TKIs can form an irreversible covalent binding to EGFR. They are pyrimidine-based compounds, and differ from quinazolines-based first-and second-generation EGFR TKIs (Table 2) [86]. Third-generation EGFR TKIs can attenuate *EGFR* T790M activity and have less epithelial toxicity due to less wild-type *EGFR* activity [86, 87]. Among them, osimertinib (AZD9291) received FDA and European Medicines Agency (EMA) approval in November 2015 and February 2016, respectively, for treatment of patients with T790M mutation-positive NSCLC after acquired resistance to first-line EGFR TKIs treatment. Table 3 shows the available efficacy data of different third-generation EGFR TKIs in clinical trials.

- Osimertinib (AZD9291)

**Table 2:** Different generations of EGFR TKIs

Generation	EGFR inhibition	Drug	Molecular Targets^a^	Adverse effect	Status
1st-generation	Reversible;	Gefitinib	*EGFR del19, L858R*	Skin rash/acne, abnormal LFT	FDA approved
	competitive	Erlotinib	*EGFR del19, L858R*		FDA approved
2nd-generation	Irreversible; covalent	Afatinib	*EGFR del19, L858R*, uncommon mutations, HER2, HER4	Diarrhea, paronychia. Skin rash	FDA approved
		Dacomitinib	*EGFR del19, L858R,* HER2, HER4	Diarrhea, skin rash/acne	Phase III
		Neratinib	*EGFR G719X*, HER2, HER4	Diarrhea, dyspnea, N/V	Phase II
3rd-generation	Irreversible;	Osimertinib	*EGFR* mutations and *T790M*	Diarrhea, skin rash	FDA approved
	covalent	Rociletinib	*EGFR T790M* mutation, IGF-1R	Hyperglycemia, QTc prolong	Withdrawn
		Olmutinib	*EGFR T790M* mutation	Diarrhea, skin exfoliation, nausea	Approved in South Korea
		ASP8273	*EGFR L858R, del19, T790M*,	Diarrhea, N/V, thrombocytopenia	Phase III Discontinued
		Nazartinib	*EGFR L858R, del19, T790M*,	Rash, diarrhea, pruritus	Phase I/II
		Avitinib (AC0010)	*EGFR L858R, del19, T790M*,	Diarrhea, skin rash, abnormal LFT	Phase I/II
		HS-10296	*EGFR* sensitive mutations *(G719X, del19, L858R, L861Q) +/−* T790M	None reported	Phase I/II
		PF-06747775	*EGFR L858R, del19, T790M*,	None reported	Phase I/II

*N/V* nausea and/or vomiting, *LFT* liver function test, *del19* deletion in exon19, *EGFR* epidermal growth factor receptor, *FDA* Food and Drug Administration

^a^The targets included FDA approved or associated targets

**Table 3:** Efficacy of third-generation EGFR TKIs in *EGFR* T790M-positive NSCLC patients

Drug	Trial	Patients (N)	Dose	ORR T790M	PFS (mo.)
Osimertinib	AURA phase I [92]	Total: 253 T790M(+): 138	20-240 mg QD	T790M(+): 61% T790M(−): 21%	T790M(+): 9.6 T790M(−): 2.8
	AURA phase I T790M(+)	63	80 mg QD	71%	9.7
	AURA phase II	210	80 mg QD	70%	9.9
	AURA phase II extension [132]	411	80 mg QD	62%	12.3
	AURA phase III [84]	416 -Osimertinib arm: 279 -Chemotherapy arm: 140		71% 31% Odds ratio:5.39 (95% CI: 3.47–8.48)	10.1 4.4 HR: 0.30 (95% CI: 0.23–0.41)
Rociletinib	TIGER-X phase I/II [98]	Total: 69 T790M(+): 51	500, 625 or 750 mg bid	45%	T790M(+): 9.6 T790M(−): 2.8
Olmutinib	HM-EMSI-101 phase I/II T790M(+) [133]	76	800 mg QD	62%	6.9
ASP8273	NCT02113813 phase I/II [134]	Total: 63 T790M(+): 58	300 mg QD	29%	6.8
Nazartinib	NCT02108964 phase I/II [105]	152	75-350 mg QD	46.9%	9.7
Avitinib (AC0010)	NCT02330367 phase I/II [106]	136	50-350 mg QD	44%	

Osimertinib (AstraZeneca, Macclesfield, UK) is an irreversible mono-anilino-pyrimidine EGFR TKI that covalently binds to the ATP-binding site, CYS797, of the EGFR tyrosine kinase domain. In EGFR recombinant enzyme assays, osimertinib showed potent activity against diverse activating *EGFR* mutations with/without T790M. According to the preclinical data, osimertinib has 200 times greater potency against L858R/T790M than wild-type *EGFR* [88]. Two circulating metabolites of osimertinib, AZ5104 and AZ7550, were detected, and both had comparable potency to sensitizing *EGFR* mutation and T790M [89]. There was no significant difference in pharmacokinetic exposure between Asian and non-Asian patients, showing a minimal food effect [90]. In addition, unlike first- and second-generation EGFR TKIs, osimertinib exposure was not affected by concurrent administration of omeprazole [91].

AURA (NCT01802632) is a phase I/II dose-escalation clinical trial of osimertinib, which enrolled 253 Asian and western NSCLC patients with acquired resistance to first- or second-generation EGFR TKIs, as defined by Jackman criteria [22, 92]. Patients were not preselected according to T790M status [92]. Thirty-one patients were treated across five dose-escalation cohorts (20, 40, 80, 160 and 240 mg oral, daily) and 222 were treated in the dose-expansion cohort.

In the dose-escalation cohort, there was no dose-limiting toxicity (DLT) and the maximum tolerated dose (MTD) has not been reached. Of the 239 evaluable patients, the objective response rate (ORR) was 51% and the disease control rate (DCR) was 84%. Patients with *EGFR*-T790M mutation had a better ORR (61% vs. 21%), DCR (95% vs. 61%), and longer median PFS (9.6 months vs. 2.8 months) than patients without an *EGFR*-T790M mutation. The drug is relatively safe, and most of the AEs were grade 1 and 2. The most common AEs were diarrhea (47%), skin toxicity (40%), nausea (22%), and anorexia (21%). When patients took higher dose levels (160 and 240 mg), there was an increasing incidence and severity of AEs (rash, dry skin, and diarrhea). Based on efficacy and safety, 80 mg daily was selected as the recommended dose for further clinical trials [92].

Then, a phase II “AURA2” study (NCT02094261) was initiated to enroll NSCLC patients with an *EGFR*-T790M mutation and acquired resistance to approved EGFR TKIs; the enrollment criteria were similar to those of the AURA study extension cohort. A preplanned pooled analysis was performed, including 201 patients from the 80 mg osimertinib expansion cohort of AURA and 210 patients from AURA2; ORR was 66%, DCR was 91%, and median PFS was 11.0 months [93].

In the phase III AURA3 study, 419 patients were randomized into osimertinib or platinum-pemetrexed chemotherapy (maintenance pemetrexed was allowed) groups after they had acquired resistance to first-line EGFR TKI therapy. The investigator-assessed PFS (primary endpoint) was significantly longer in the osimertinib arm than in the chemotherapy arm (median 10.1 vs. 4.4 months; HR 0.30; *p* < 0.001). The FDA has granted regular approval to the third-generation EGFR TKI, osimertinib, for the treatment of patients with metastatic *EGFR* T790M mutation-positive NSCLC.

In the preclinical study, osimertinib demonstrated greater penetration of the mouse blood-brain barrier than gefitinib, rociletinib, or afatinib [94]. There were several reports of dramatic intracranial response to osimertinib in patients with *EGFR* T790M lung cancer [94, 95]. A phase I study (BLOOM, NCT02228369), which has enrolled pretreated *EGFR*-mutant NSCLC patients with leptomeningeal metastasis treated with 160 mg osimertinib once daily, is ongoing. The preliminary data is promising [96].

- Rociletinib (CO-1686)

Rociletinib, a 2,4-disubstituted pyrimidine compound, is an oral, irreversible, mutant-selective inhibitor of activating *EGFR* mutations, including T790M, and spares wild-type *EGFR* [97]. TIGER-X (NCT01526928A), a phase I/II trial of rociletinib, enrolled 130 *EGFR*-mutant NSCLC patients with acquired resistance to first- or second-generation EGFR TKIs [83]. The ORR was 59% for the 46 evaluable T790M mutation-positive patients and 29% for the 17 T790M mutation-negative patients [83]. Because of targeting of IGF-1R, hyperglycemia (22%) was detected as the most common grade 3 AE. An independent updated analysis of the TIGER-X trial showed that the T790M mutation-positive patients had an ORR of 45% [98]. In addition, a series of cases with response to osimertinib after resistance to rociletinib were reported [99]. Clovis Oncology, Inc. decided to stop enrollment in all ongoing rociletinib studies and terminate the future development program in May 2016.

- Olmutinib (BI-1482694/HM61713; Olita™)

A phase I/II dose escalation clinical trial, HM-EMSI-101 (NCT01588145), was initiated in South Korea [100]. Patients took olmutinib in doses ranging from 75 to 1200 mg/day. Among the 34 patients with NSCLC harboring T790M detected by a central laboratory, the ORR was 58.8%. The DCR was 97.1% for patients treated with olmutinib in doses greater than 650 mg. The most common DLTs involved gastrointestinal symptoms, abnormal liver function (AST/ALT), and increasing amylase/lipase levels. Therefore, 800 mg/day was selected as the recommended phase II dose. Seventy-six patients with centrally confirmed T790M mutation-positive NSCLC were enrolled in part II of the study, and 70 were evaluable for response. The ORR was 61% and median PFS was 6.9 months [101]. Based on the aforementioned result, olmutinib was first approved in South Korea in 2016. However, Boehringer Ingelheim decided to stop the co-development of this drug because of an unexpected grade 3/4 skin toxicity (including palmoplantar keratoderma) [102].

- ASP8273

Preclinical data showed ASP8273 had antitumor activity against EGFR TKI-resistant cells, including those with resistance to osimertinib and rociletinib [103]. A multi-cohort, phase 1 study (NCT02113813) was initiated to evaluate the safety and efficacy of ASP8273 in NSCLC patients with disease progression after EGFR TKI treatment. The most common AEs included diarrhea (47%), nausea (42%), and fatigue (32%). The most common grade 3/4 AE was hyponatremia (17%). Across all doses, the ORR was 30.7%, and median PFS was 6.8 months in patients with *EGFR* T790M [104]. A phase III randomized clinical trial (SOLAR) was conducted to compare the efficacy and safety of ASP8273 with that of erlotinib or gefitinib as first-line treatment for advanced *EGFR*-mutant NSCLC (NCT02588261). However, Astellas Pharma (OTCPK: ALPMY) terminated the phase III SOLAR study in May 2017 because the treatment advantage apparently was not adequate enough to justify continuation.

- Nazartinib (EGF816)

A phase I/II first-in-human study, NCT02108964 (EGF816X2101), investigated nazartinib in *EGFR*-mutant patients. A total of 152 patients were treated across seven cohorts using doses ranging from 75 to 350 mg [105]. Among the 147 evaluable patients, the ORR and DCR were 46.9% and 87.1%, respectively. The median PFS across all dose cohorts was 9.7 months. Skin rash (54%), diarrhea (37%), and pruritus (34%) were the most common AEs. The skin rashes related to nazartinib were different from those caused by other EGFR TKIs in pattern, location, and histology. The most common grade 3/4 AE was diarrhea (16%) [105]. A phase II clinical trial with six cohorts is ongoing. In addition, a phase Ib/II trial (NCT02335944 and NCT02323126) is ongoing to investigate the efficacy of combined treatments with INC280, a specific MET inhibitor, and with nivolumab, an anti-PD-1 monoclonal antibody in patients with *EGFR*-T790M mutation after acquired resistance to first-line EGFR TKI.

- AC0010

A phase I/II, first-in-human dose-escalation and expansion phase clinical trial (NCT02330367) was carried out with advanced NSCLC patients with acquired T790M mutation after first-generation EGFR TKIs treatment [106]. In all, 136 patients have been treated across seven cohorts (50, 100, 150, 200, 250, 300, and 350 mg BID), and MTD has not been reached. The most common drug-related AEs were diarrhea (38%), rash (26%) and ALT/AST elevation. Grade 3/4 AEs of diarrhea (2%), rash (2%) and ALT/AST elevation (4%, 2%) were recorded. The 124 evaluable patients had ORR and DCR of 44% and 85%, respectively. Because of the drug safety profile and activity against NSCLC with acquired T790M mutation, a phase II, AEGIS-1 study is ongoing to evaluate treatment efficacy for patients with T790M mutation-positive NSCLC with acquired resistance to first-generation EGFR TKIs. An open label, randomized phase III trial (NCT03058094) also is ongoing to compare AC0010 (300 mg, BID) with pemetrexed/cisplatin (4–6 cycles) in patients with advanced NSCLC who have progressed following prior therapy with EGFR TKI. T790M in biopsy samples was confirmed by a central laboratory.

- HS-10296

An open-label, multicenter, phase I/II dose escalation and expansion trial (NCT02981108) is currently recruiting patients with locally advanced or metastatic NSCLC after acquired resistance to first- and/or second-generation EGFR TKIs.

- PF-06747775

PF-06747775 has potent antitumor efficacy against NSCLC harboring a classical mutation with/without T790M. It significantly attenuates T790M activity and has less toxicity because of the reduction of proteome reactivity relative to earlier EGFR TKIs [107, 108]. A phase I/II clinical trial (NCT02349633) involving patients with advanced NSCLC harboring *EGFR* mutations (Del19 or L858R with/without T790M) is ongoing.

#### Combination therapy

- *Vertical pathway*

Cetuximab is a recombinant human/mouse chimeric EGFR IgG1 monoclonal antibody. Combining afatinib and cetuximab may be useful for patients who have progressed after receiving EGFR TKI therapy and chemotherapy [109]. Among 126 patients, the response rate of patients with T790M-positive and T790M-negative tumors was comparable (32% vs. 25%; *p* = .341). The two groups showed no statistical difference in PFS. The NCCN Panel recommends considering an afatinib/cetuximab regimen for patients who have progressed after receiving EGFR TKIs and chemotherapy [48]. However, skin rash (90% all grades) and diarrhea (71% all grades) were the two most common adverse effects. Grades 3 and 4 adverse effects were 44% and 2%, respectively. Because of the high rate of AEs with this combination therapy, it is no longer a preferred treatment for patients with tumor harboring *EGFR* T790M mutations [110].

- *Horizontal pathway*

Since bypass signaling pathway activation is an important acquired resistance mechanism of EGFR TKIs, it is reasonable to combine inhibition of EGFR pathway signaling and inhibitors for the bypass signaling pathway to overcome resistance. Different horizontal combination strategies are being investigated, but results are preliminary and immature (Table 4).

**Table 4:** Main mechanisms involved in acquired resistance to EGF receptor-tyrosine kinase inhibitors and the associated targetable drugs

Molecular alteration	Pathway	Targetable drug
HER2 amplification		Afatinib, Trastuzumab, ado-trastuzumab emtansine (TDM1)
MET overexpression/genetic alteration		● Anti-HGF antibody: Rilotumumab, Ficlatuzumab ● Anti-c-MET antibody: MET Mab, Emibetuzumab (LY2875358) ● Selective c-MET inhibitor: Tivantinib (ARQ197), Capmatinib (INC280), Savolitinib (AZD6094), Tepotinib (EMD 1214063), SGX523, SAR125844, ● Multikinase inhibitors: Crizotinib, Cabozantinib (XL184), Glesatinib (MGCD265), Merestinib (LY2801653), S49076
PIK3CA	PI3K-AKT-mTOR	● PI3K inhibitor: Pilaralisib (XL147), Dactolisib (BEZ235) and Pictilisib (GDC-0941), Buparlisib (BKM120) ● AKT inhibitor: MK-2206 ● mTOR inhibitor: Everolimus, Temsirolimus, Ridaforolimus
BRAF	Ras-Raf-MEK-ERK	Vemurafenib (PLX4032), Dabrafenib (GSK2118436), Selumetinib, LY3009120
AXL overexpression	GAS6-AXL	● Tyrosine kinase inhibitor: Cabozantinib (XL 184) ● AXL antibody: E8, D9, Mab173 ● AXL decoy receptor: AXL-Fc, MYDI

*MET* amplification is an important mechanism of acquired resistance to EGFR TKI therapy [31, 111]. A randomized, open-label, phase 2 study enrolled patients with advanced NSCLC (enriched for *EGFR*-mutant disease) who developed acquired resistance to erlotinib to receive emibetuzumab (LY2875358), a humanized IgG4 monoclonal bivalent MET antibody, with or without erlotinib therapy. The ORR of patients whose re-biopsy samples harbored MET overexpression (≥60%) was 3.8% in the combination arm and 4.8% in the monotherapy arm [112]. In Japan, another phase II clinical trial enrolled 45 patients with advanced *EGFR*-mutant NSCLC who developed acquired resistance to first-generation EGFR TKIs to receive tivantinib (ARQ197) and erlotinib combination therapy. The response rate was 6.7%. High MET expression (≥ 50%) was detected by immunohistochemical stain in 48.9% of the patients, including all three partial responders [113]. In addition, a combination of capmatinib (INC280) and gefitinib was tested in a phase 2 study (NCT01610336) in *EGFR*-mutant NSCLC patients after acquired resistance to gefitinib. *EGFR* T790M NSCLCs were excluded and high cMET expression was required. Of the 65 evaluable patients, the ORR was 18% and DCR was 80%. More responses were seen in tumors with *MET* amplifications [114].

In addition to *MET* amplification, different medications are being investigated to inhibit other bypass signaling pathways, including a heat shock protein 90 inhibitor, AUY922 (ClinicalTrials.gov: NCT01259089 and NCT01646125); a JAK inhibitor, ruxolitinib (ClinicalTrials.gov: NCT02155465 and NCT02145637); a MET/AXL/FGFR inhibitor S- 49076 (EU Clinical Trials Register: EudraCT Number: 2015–002646-31) and a PI3K inhibitor, buparlisib (BKM120) (ClinicalTrials.gov: NCT01570296 and NCT01487265).

Furthermore, combination therapy with osimertinib has been investigated. The TATTON study (ClinicalTrials.gov: NCT02143466) enrolled patients who received osimertinib-based combination therapy with either a MET inhibitor (savolitinib), MEK inhibitor (selumetinib), or anti-PD-L1 monoclonal antibody (durvalumab) [115]. However, the rate of drug-related interstitial disease was high in the osimertinib plus durvalumab arm, so the development of this combination therapy was discontinued [116]. Other clinical trials, including osimertinib in combination with ramucirumab, necitumumab, bevacizumab, or navitoclax (ClinicalTrials.gov, NCT02789345, 02496663, 02803203 and 02520778), are ongoing.

Combination therapies have higher rates of toxicities and side effects than a single agent does. Although the aforementioned medications have been evaluated in clinical trials, clinicians should keep in mind the possibility of AEs when prescribing combination therapy.

#### Immunotherapy

For subsequent therapy, or immunotherapy, nivolumab and pembrolizumab have been approved as standard treatment, and high-level PD-L1 expression in tumors can predict a higher response rate. Phase III trials assessing pembrolizumab, nivolumab, or atezolizumab compared to docetaxel as subsequent therapy for patients with metastatic NSCLC found there were no survival benefits for *EGFR*-mutant lung cancer patients. Also, there were not enough patients with these mutations to determine whether there were statistically significant differences. However, immunotherapy was comparable to chemotherapy and was better tolerated. [117–119]. Until now, there is not enough evidence to recommend pembrolizumab, nivolumab, or atezolizumab as subsequent therapy for *EGFR*-mutant patients.

In vitro, *EGFR*-mutant lung cancer cells inhibited antitumor immunity by activating the PD-1/PD-L1 pathway to suppress T-cell function [120]. This finding indicates that EGFR functions as an oncogene through cell-autonomous mechanisms and raises the possibility that other oncogenes may drive immune escape [120]. However, retrospective studies showed that NSCLCs harboring *EGFR* mutations were associated with low response rates to PD-1/PD-L1 inhibitors, which may have resulted from low rates of concurrent PD-L1 expression and CD8(+) TILs within the tumor microenvironment [119]. A retrospective study on the efficacy of nivolumab in patients with *EGFR* mutation-positive NSCLC after EGFR TKI failure found that T790M-negative patients were more likely than T790M-positive patients to benefit from nivolumab [121].

Different phase 1 trials combining EGFR TKIs with immunotherapies include nivolumab (ClinicalTrials.gov, number NCT01454102); pembrolizumab (ClinicalTrials.gov, number NCT02039674); and atezolizumab (ClinicalTrials.gov, number NCT02013219). These studies are all ongoing.

## Conclusions

EGFR TKIs are currently the standard first-line treatment of patients with advanced NSCLC harboring activating *EGFR* mutations. After acquiring resistance to first-line EGFR TKI therapy, it is important that the mechanisms of acquired resistance in all patients are explored. Then, based on the mechanism, subsequent treatment can be chosen. Continuation of EGFR TKI therapy is suitable for select patients with asymptomatic progression and/or oligoprogression. Repeat tumor biopsy to detect the *EGFR* T790M mutation is the current standard of care, and osimertinib has been approved for patients with acquired *EGFR* T790M-mutant disease. Liquid biopsy is an alternative method to detect plasma *EGFR* T790M mutation and to identify patients suitable for osimertinib therapy. Combination therapy may be effective for acquired resistance resulting from activation of the bypass signaling pathway. Advances in the detection method for different resistance mechanisms and the development of new drugs are both urgently needed for personalized therapy.

## Abbreviations

95% CI: 95% confidence interval
AEs: adverse effects
ASCO: American Society of Clinical Oncology
ctDNA: circulating tumor DNA
DLT: dose-limiting toxicity
EGFR: epidermal growth factor receptor
EMT: epithelial–mesenchymal transition
ESMO: European Society for Medical Oncology
FDA: Food and Drug Administration
MLT: maximum tolerated dose
NCCN: National Comprehensive Cancer Network
NSCLC: non-small cell lung cancer
ORR: objective response rate, DCR disease control rate
OS: overall survival
PD: progressive disease
PFS: progression-free survival
SCLC: small cell lung cancer
TKI: tyrosine kinase inhibitor
